# Quality of life and its association with predictors in lung transplant recipients: a latent profile analysis

**DOI:** 10.3389/fpubh.2024.1355179

**Published:** 2024-04-29

**Authors:** Liqin Song, Qing Luo, Chunqin Liu, Ying Zhou, Danxia Huang, Chunrong Ju, Huifang Chen, Thomas Kwok Shing Wong, Jiani Chen, Wenying Tan, Chuyuan Miao, Yu Ma, JingWen Chen

**Affiliations:** ^1^School of Nursing, Guangzhou Medical University, Guangzhou, China; ^2^Department of Nursing, National Center for Respiratory Medicine, Guangzhou Institute of Respiratory Health, The First Affiliated Hospital of Guangzhou Medical University, Guangzhou, China

**Keywords:** quality of life, lung transplant recipients, latent profile analysis, mindfulness, optimism, nursing

## Abstract

**Backgrounds:**

Improving quality of life (QOL) is one of the main aims of lung transplantation (LTx). There is a need to identify those who have poor quality of life early. However, research addressing inter individual quality of life variability among them is lacking. This study aims to identify group patterns in quality of life among lung transplant recipients and examine the predictors associated with quality of life subgroups.

**Methods:**

In total, 173 lung transplant recipients were recruited from one hospital in Guangdong Province between September 2022 and August 2023. They were assessed using the Lung Transplant Quality of Life scale (LT-QOL), Mindful Attention Awareness Scale (MAAS), Life Orientation Test-Revised scale (LOT-R), and Positive and Negative Affect Scale (PANAS). Latent profile analysis was used to identify QOL subtypes, and logistic regression analysis was used to examine the associations between latent profiles and sociodemographic and psychosocial characteristics.

**Results:**

Two distinct QOL profiles were identified: “low HRQOL” profile [*N* = 53 (30.94%)] and “high HRQOL” profile [*N* = 120 (69.06%)]. Single lung transplant recipients, and patients who reported post-transplant infection, high levels of negative emotion or low levels of mindfulness and optimism were significantly correlated with the low QOL subgroup.

**Conclusion:**

Using the domains of the LT-QOL scale, two profiles were identified among the lung transplant recipients. Our findings highlighted that targeted intervention should be developed based on the characteristics of each latent class, and timely attention must be paid to patients who have undergone single lung transplantation, have had a hospital readmission due to infection, exhibit low levels of optimism, low levels of mindfulness or high negative emotions.

## Introduction

Lung transplantation (LTx) has become the most effective treatment and a life-saving intervention for patients with end-stage lung and pulmonary vascular diseases (1). According to the International Society for Heart and Lung Transplantation (2), the number of lung transplantation has steadily increased in recent years; as of 2021, approximately 70,000 patients have received LTx transplants worldwide and the 1-year conditional survival rate for LTx patients has reached 90%, with a median survival of approximately 6 years. With a markedly improved survival of patients through recent decades, improving the quality of life (QOL) is becoming one of the key clinical aims (3, 4). QOL, as a comprehensive construct, involves physical health, psychological state, personal beliefs, social relationships and their relationships to salient features of their environment (5). One study found that LTx recipients’ long-term physical QOL was substantially poorer than normative sample levels and even poorer than it had been earlier posttransplant; and it worsened over time when complications arose (6). Furthermore, LTx patients reported significantly worse QOL when compared to those heart and liver transplantation recipients (7). Additionally, patients who have lower QOL may report more negative clinical and psychological outcomes. Specifically, lower QOL related-indicators were associated with a higher risk of all-cause mortality and unplanned readmission (8, 9). Conversely, those outcomes were negatively associated with QOL, creating a vicious cycle that ultimately may lead to a decreased life expectancy (10). Hence, ongoing QOL evaluation is required, and it is necessary to identify patients with poor QOL to provide supportive care (11).

For LTx recipients, achieving and maintaining satisfactory QOL is an intricate and ongoing process influenced by myriad factors. Several studies have demonstrated that age, marital status, disease diagnosis, and transplant type may associate with QOL after transplantation (12, 13). Furthermore, a well-established association exists between emotions and QOL in patients. The possible reason is that emotions can impact breathing patterns, and influence the autonomic nervous system’s control over respiration (14), exerting detrimental effects on the QOL of LTx recipients (15). Considering that mood states are more easily recognized and management, this study focused on the possible effects of positive and negative emotions on QOL. Moreover, recent advancements in positive psychology have generated fresh insights into the relationship between individual variations in QOL and the positive dimensions of human potential, strengths, and resources, which consequently garner growing interest and attention within the academic community. Several positive psychological constructs have emerged as advantageous within the context of QOL among organ transplant patients (14, 16). One such construct is optimism, which is characterized by the tendency to expect positive outcomes across various life domains and the ability to mediate stress through perceived positive or negative emotions (17). Recent research suggests that the higher the optimism level, the higher the QOL. Both cross-sectional and long-term follow-up studies of patients diagnosed with cancer have revealed that compared to patients with a pessimistic disposition, those with an optimistic personality reported better QOL across several domains, including emotional functioning, social functioning, pain, and body image (18–20). Mindfulness, another internal psychological trait rooted in traditional Buddhism, refers to the nonjudgmental and intentional awareness of experiences in the present moment (21). Mindfulness is positively correlated with numerous health-related variables, such as QOL and optimistic emotions, and inversely correlated with negative mental symptoms, such as depression and anxiety (22). Nevertheless, there has been a lack of research concerning the contribution of positive psychological constructs (e.g., optimism and mindfulness) to QOL and its specific subgroups among LTx patients. As such, exploring the factors affecting the QOL of the LTx recipient is becoming an increasingly crucial research topic that needs resolution.

A review of the available literature found that most extant scholarly investigations about the QOL of LTx patients have traditionally employed aggregate scores or critical cutoff values as the primary means of assessment (7, 23). Such an approach overlooks the intricate interplay of individual-level factors and fails to harness the rich, nuanced information embedded within each study participant. However, latent profile analysis (LPA), as a person-centered technique, enables researchers to identify latent subgroups or profiles within a particular population, revealing unexplored patterns and uncovering previously unconsidered nuances (24), thereby offering insights essential for tailored interventions, precise classifications, and a deeper comprehension of the heterogeneity among individuals (25). Several studies have identified distinct QOL profiles in different populations. For instance, one Romanian study identified three QOL subgroups using the World Health Organization Quality of Life-Brief (WHOQOL-BREF) among the patients 65 years of age and older from Bucharest on the geriatric ward (26). Another similar study (27) used the Stoma Quality of Life Scale (SQOL) to identify four profiles for patients with a stoma. Hence, the application of LPA could potentially identify homogeneous subgroups of QOL, which may enable us to gain an enhanced understanding of QOL patterns and the differences within these patterns among LTx recipients. In addition, non-specific QOL measurements were most applied in prior studies, such as the 36-Item Short Form Health Survey (SF-36) (28) and WHOQOL-BREF (29), which may lack the sensitivity to detect subtle changes or issues specific to LTx recipients because they are facing unique challenges and health-related issues. However, the Lung Transplant Quality of Life (LT-QOL) (30), a dedicated QOL scale tailored to this specific population, has effectively addressed this concern. It can accurately capture these nuances, ensuring the assessment’s relevance and appropriateness.

Over the past few years, lung transplantation in China has developed rapidly. According to relevant research, a total of 2,801 lung transplantations have been performed between 2015 and 2021 (31). However, due to the complexity of perioperative management and the late development of China’s lung transplantation system, compared to other transplant populations, there are fewer studies about QOL among LTx patients, particularly for applying the LPA. Above all, this study seeks to identify subgroups with similar patterns of QOL in a sample of Chinese LTx patients using the LT-QOL, and determines whether demographic characteristics, clinical characteristics, and psychological factors (e.g., mindfulness and optimism) are associated with specific profile membership of QOL. By achieving these objectives, it should contribute valuable knowledge to the fields of transplant medicine and psychology, ultimately enhancing the QOL and overall well-being of LTx recipients in the Chinese context.

## Methods

### Participants and procedures

A cross-sectional study was conducted at the First Affiliated Hospital of Guangzhou Medical University between September 2022 and August 2023. To be included in the study, patients had to (1) have undergone LTx with clinical stability, (2) be 18 years of age or older, (3) be conscious, (4) be able to communicate in Chinese, and (5) be willing to participate in the study. Patients who have undergone regrafting or combined organ transplantation were excluded. In this study, no organs from political prisoners, executed prisoners, religious minorities, or other persecuted groups were used. Informed consent was obtained from all individual participants included in the study. A total of 175 questionnaires were distributed: two could not be included due to invalid data. Finally, the data from 173 patients were valid, with an effective response rate of 98.9%. Participants received some gifts as compensation for their participation. This study was approved by the Ethics Committee of Guangzhou Medical University (L202210008).

### Data collection

Three researchers collaborated to collect data, having undergone unified training in survey methodologies, including explaining survey items and providing clarifications as needed. Prior to the formal investigation, a preliminary survey was conducted to ensure the questionnaire’s clarity and ease of understanding. With the support of medical staff in the lung transplantation department, patients in clinical follow-up period post-transplantation were recruited from WeChat patient groups. Subsequently, the researchers screened volunteers who met the inclusion criteria. The survey was conducted in the follow-up outpatient department using a one-on-one on-site questionnaire collection method. During data collection, the researchers provided detailed explanations of the research purpose and significance, obtained participants’ consent, and offered assistance in understanding the questions as needed. Upon completion, the researchers immediately conducted an on-site double-check to ensure the questionnaire’s effective retrieval.

## Measures

### Demographic and clinical characteristics

Age, gender, education level, marital status, monthly income (Chinese yuan), height (m), weight (kg) and disease-related data were included. Body Mass Index (BMI) was computed using the formula of kg/m^2^. Disease-related data were collected from electronic files, including diagnosed disease, time since lung transplant, the duration of the intensive care unit (ICU) stay, type of operation, length of post-transplant hospital stays, use of ECMO, unplanned readmissions within 30 days, major post-op complications (infection and rejection) that caused readmission, and major preoperative comorbidities (hypertension and diabetes). ECMO was used for extracorporeal respiratory and circulatory support.

### Quality of life

QOL was assessed by the Chinese version of the Lung Transplant Quality of Life (LT-QOL) (30). The 60-item original multidimensional instrument developed by Singer et al. in 2019 contains a total of four subscales with 10 dimensions: as symptoms (pulmonary, GI, and neuromuscular), health perceptions (worry about future health and treatment burden), functioning (cognitive limitations and sexual problems), and well-being (anxiety/depression, health distress, and general quality of life). The Chinese version (32) contains 40 items in three subscales with 11 dimensions: symptoms (pulmonary, cough, swallow, appetite, gastric, and diarrhea), well-being (worry about future health, anxiety, and general quality of life), and function (sexual problems and cognitive limitations). Each item was scored on a scale from 1 to 5 points, with a total score ranging from 40 to 200. A higher total score indicates a better QOL. Cronbach’ s α was 0.95 in this study.

### Mindfulness

Mindfulness was measured using the Chinese version of the Mindful Attention Awareness Scale (MAAS) (33), a single-dimension scale consisting of 15 items measured using a 6-point Likert scale (1 = “almost always,” 6 = “almost never”). The total score on the scale ranges from 15 to 90, with a higher score indicating a higher level of individual mindfulness. Cronbach’s α was 0.77 in this study.

### Optimism

Optimism was assessed using the Chinese version of the Life Orientation Test-Revised (LOT-R) (34). This scale has 6 content items, 3 assessing positive expectations and 3 assessing negative expectations. All responses used a 5-point Likert scale (0 = “strongly disagree,” 4 = “strongly agree”). Before the analysis, negatively worded items were reverse coded. Higher scores reflect greater levels of optimism. Cronbach’s α was 0.80 in this study.

### Positive and negative affect

Emotional predisposition was assessed using the Chinese version of the Positive and Negative Affect Scale (PANAS) (35). This scale has 20 content items with two subscales, 10 assessing positive affect and 10 assessing negative affect. All questions were rated on a 5-point Likert scale (1 = “not at all,” 5 = “extremely”), referring to the preceding 2 weeks. Higher scores indicate higher positive and negative affect. Cronbach’s α were both 0.86 for the PANAS-P and PANAS-N in this study.

### Statistical analysis

Statistical analyses were performed using *Mplus* 8.3 and *SPSS* 26.0. First, with *Mplus Version 8.3*, a latent profile analysis was conducted using the mean scores of the 11 dimensions of QOL to identify the classes of the lung transplant recipients’ QOL. LPA is a person-centered approach that helps to identify latent subgroups of individuals on the basis of patterns in the means of the observed variables (36). To assess the statistical values of the model, the Akaike information criterion (AIC), Bayesian information criterion (BIC), sample-size adjusted BIC (SABIC), entropy (Entropy), Lo–Mendell–Rubin Test (LMRT), and the Bootstrap Likelihood Ratio Test (BLRT) were reported. Generally, lower AIC, BIC, and SABIC values indicated a better absolute fit. The LMRT and BLRT *p*-values were employed to compare the fitted discrepancy between the two models, with a significant *p*-value suggesting that a model with *k* classes described the data better than one with *k-1* classes. The entropy value ranges from 0 to 1, with scores closer to 1 indicating that the classification is accurate and sound (ideally, above 0.70).

Additionally, after determining the optimal model, a one-way analysis of variance (ANOVA) was performed to examine whether the variables distinguished the QOL classes. Logistic regression was applied to examine whether the indicators with statistical significance independently predicted the latent profiles.

## Results

### General characteristics

Of the 173 patients, 139 were males and 34 were females. The mean age was 56.4 (SD = 12.7) years. Most were married (90.8%) and had a college education (39.9%). Seventy percent of the patients within 3 years after lung transplantation. More Socio-demographic and clinical characteristics are displayed in Table 1. The bivariate correlations among the LT-QOL are presented in Supplementary Table S1, showing that variables included in LPA are independent (all correlations were below 0.60).

**Table 1:** Participants’ sociodemographic and clinical characteristics (*N* = 173).

	*M* ± SD or *n* (%)
Age (years)	56.4 ± 12.7
**Gender**	
Male	139 (80.3%)
Female	34 (19.7%)
**Education level**	
Primary school or below	22 (12.7%)
Junior	47 (27.2%)
High school and technical secondary school	35 (20.2%)
College or above	69 (39.9%)
**Marital status**	
Married	168 (90.8%)
Unmarried	5 (9.2%)
**Monthly income (Chinese Yuan)**	
<3,000	24 (13.9%)
3,000–5,999	56 (32.4%)
6,000–8,999	37 (21.4%)
9,000–11,999	20 (11.6%)
≥12,000	36 (20.8%)
**Diagnostic indication for transplant**	
Pulmonary fibrosis	74 (42.8%)
Obstructive lung disease	49 (28.3%)
Others	50 (28.9%)
**Time since lung transplant**	
<3 months	15 (8.7%)
3–6 months	17 (9.8%)
6 months–1 year	16 (9.2%)
1–3 years	74 (42.8%)
>3 years	51 (29.5%)
**Type of operation**	
Single-lung transplantation	68 (39.3%)
Bilateral lung transplantation	105 (60.7%)
BMI (kg/m^2^)	21.4 ± 3.6
**Use of ECMO**	
Yes	64 (37.0%)
No	109 (63.0%)
**Unplanned readmissions within 30 days**	
No	136 (78.6%)
Yes	37 (21.4%)
**Post-transplant infection**	
No	73 (42.2%)
Yes	100 (57.8%)
**Post-transplant rejection**	
No	131 (75.7%)
Yes	42 (24.3%)
**Preoperative hypertension**	
No	148 (85.5%)
Yes	25 (14.5%)
**Preoperative diabetes**	
No	148 (85.5%)
Yes	25 (14.5%)
length of post-transplant hospital stays (days)	31.4 ± 15.2
The duration of ICU stays (days)	16.3 ± 17.8
Mindfulness	56.7 ± 9.5
Optimism	18.6 ± 3.7
Positive emotion	32.6 ± 6.9
Negative emotion	19.0 ± 6.2

### Latent profiles analysis of QOL

The model fit indices for each possible latent profile are presented in Table 2. Within our study, we stopped analyzing models at more than five profiles. Because, for solutions with five or more profiles, the smallest profiles comprised less than 5% of the total sample, indicating profiles of this size may be spurious (37, 38). For the AIC, BIC, and SABIC, the values decreased when the number of profiles increased. For all the estimated models, the entropy values were above 0.80, indicating that all models provided fairly accurate classifications. While regarding LMRT likelihood ratio tests, *p*-values were only significant in the default one-profile model and two-profile model when BLRT tests were all significant (*p* < 0.001). Moreover, compared to the one-class model, the two-profile solution had lower AIC, BIC, and SABIC values. In addition, the probabilities for the classification of patients into their respective models were all above 0.9, indicating credible delineation of the profiles. Moreover, classification quality, based on the average latent profile probabilities for most likely class membership, suggested that the 2-classes had a good discriminability and reliable classification: 0.969 for latent class 1 and 0.982 for latent class 2. In summary, the two-class model was finally selected as the best representation of the data.

**Table 2:** Model fit indexes of LPA.

Models	AIC	BIC	aBIC	Entropy	LMRT	BLRT	Smallest profile (%)	Posterior probability				
								1	2	3	4	5
1	4685.00	4754.38	4684.71	NA	NA	NA	NA	NA				
2	**4241.41**	**4348.62**	**4240.96**	**0.92**	**<0.001**	**<0.001**	**30.94**	**0.97**	**0.98**			
3	4143.00	4288.05	4142.39	0.87	0.20	<0.001	12.14	0.97	0.92	0.94		
4	4107.43	4290.32	4106.66	0.89	0.65	<0.001	11.09	0.99	0.94	0.96	0.89	
5	4040.19	4260.92	4039.26	0.91	0.62	<0.001	2.88	0.99	0.98	0.89	0.99	0.94

Boldface indicates the selected model. NA, not applicable; AIC, Akaike Information Criteria; BIC, Bayesian Information Criteria; aBIC, Adjusted Bayesian Information Criteria; LMRT, Vuong-Lo–Mendell–Rubin likelihood ratio test; BLRT, Bootstrapped Likelihood Ratio Test; Smallest profile (%), Smallest percentage of each class.

### Latent profile characteristics

**Figure 1:**
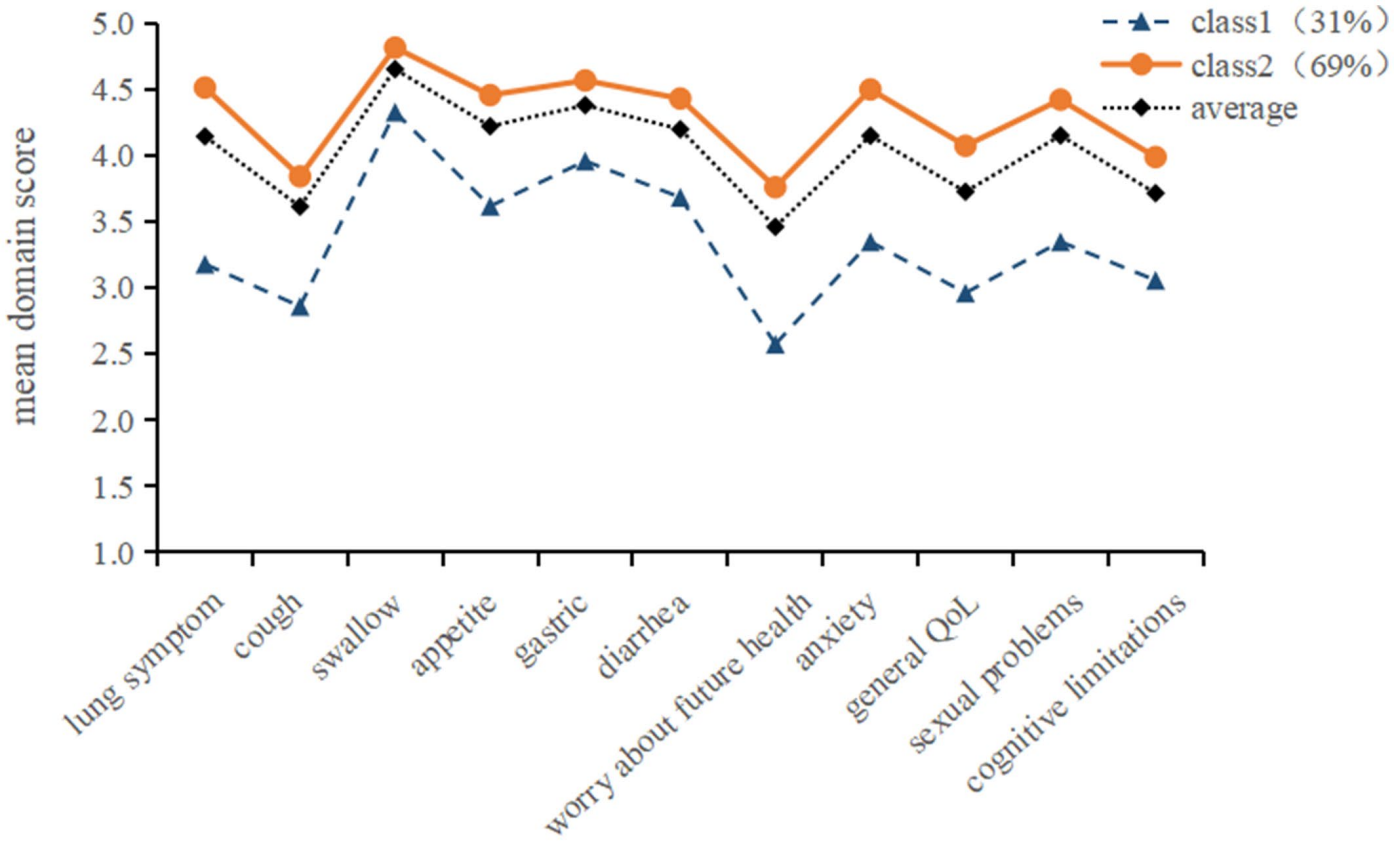
Latent profile model of quality of life in lung transplant recipients. Class1 = Low QOL; Class2 = High QOL.

The mean scores of the two profiles on the 11 domains of the LT-QOL are shown in Figure 1. Class 1, which was labeled “low QOL” profile, had a lower number of patients (31%, *n* = 53) and was characterized by low QOL scores for most of the domains considered (mean = 3.34). Class 2 accounted for 69% (*n* = 120) of the participants. And the mean values (mean = 4.35) were higher than the average scores for the entire sample (mean = 4.04). We labeled this profile “high QOL.”

### Differences in the variables between the latent profiles of QOL

The Chi-squared test and Analysis of Variance (ANOVA) test showed that significant differences between profiles in terms of BMI, Infection, Type of operation, Optimistic, Mindfulness, Negative and Positive emotions (*p* < 0.1), as shown in Supplementary Table S2. Table 3 presents the results of the logistic regression analysis. Specifically, with “low QOL” being used as the reference category within the analysis, compared with single-lung transplant recipients, bilateral-lung transplant recipients were more likely to fall in the profile “high QOL” (OR = 3.09, 95%CI = 1.28–7.43). LTx patients required readmission due to infection were more likely to belong to the “low QOL” profile (OR = 0.31, 95%CI = 0.12–0.79). As expected, higher optimism and mindfulness scores (OR = 1.15, 95%CI = 1.02–1.31, OR = 1.09, 95%CI = 1.03–1.15) were related to classification as a member of the “high QOL” profile rather than the “low QOL” profile. Higher levels of negative emotion were more likely to be classified into the “low QOL” profile, while positive emotion did not show significant differences between the classes.

**Table 3:** Variables associated with profile membership.

Variables	β	SE	p	OR (95%CI)
Type of operation (reference: single)	**1.11**	**0.47**	**0.008**	**3.47 (1.39, 8.67)**
BMI	0.06	0.06	0.360	1.06 (0.94, 1.19)
Infection (reference: no infection)	**−1.17**	**0.48**	**0.014**	**0.31 (0.12, 0.79)**
Optimism	**0.13**	**0.07**	**0.040**	**1.14 (1.00, 1.30)**
Mindfulness	**0.08**	**0.03**	**0.009**	**1.08 (1.02, 1.15)**
Negative emotion	**−0.10**	**0.04**	**0.013**	**0.91 (0.84, 0.98)**
Positive emotion	0.03	0.04	0.410	1.03 (0.96, 1.12)

Boldface indicates the significantly variables (*p* < 0.05).

## Discussion

To the best of our knowledge, this is the first study of Chinese LTx recipients to examine the potential predictors and correlates of both QOL and positive psychological variables via the LT-QOL. Our study yields two key findings. First, it identified two distinct latent profiles of QOL among LTx patients, namely, “Low QOL” profile and “High QOL” profile. Second, we determined five factors that were associated with QOL profile: bilateral lung transplantation, patients who were not hospitalized for infection, higher levels of optimism and mindfulness, and a low level of negative emotions, which were positively associated with the high QOL profile. The identification of these predictors serves as a valuable step toward understanding the varying profiles of QOL among lung transplant recipients. These findings could facilitate more efficient clinical resource allocation and care planning, helping clinicians formulate individualized and precisely tailored intervention strategies aimed at enhancing the QOL of LTx patients.

Our findings revealed that LTx patients using the LT-QOL scale reported a moderate level of QOL, which was similar with previous studies (13, 39, 40). Although the tools used in measuring QOL and the study methodologies were varied, LTx provided improvements in QOL for the most patients relative to their pre-transplant baseline in most studies. For instance, one longitudinal study conducted by Singer et al. using four different scales to assess QOL (41), had found that LTx significantly enhanced QOL and the improvements were sustained over a long period after surgery. This finding has also been supported in some reviews (42, 43). Nevertheless, a few studies showed some patients were at a low level of QOL after LTx, which were likely due to the interaction of various factors including physical, psychological, and social factors (11, 44). Our study mainly focused on identifying the LTx patients at risk for a low QOL using LPA, and determined the multidimensional predictors associated with the specific profile membership of QOL.

Of the two profiles identified, the largest was the high QOL profile, to which 69.0% of LTx patients belonged. It was characterized by the highest level in all LT-QOL domains, suggesting that transplantation has shown an advantage in improving QOL. The second profile was named low QOL profile, accounting for 31.0% of patients, as these participants reported a low level of all QOL areas. These results indicated that lung transplantation recipients’ perceptions of QOL varied, exhibiting individual differences. It is worth noting that “low QOL” profile had the lowest scores for the “cough” and “worry about future health” domains. One probable reason is that the main complications after lung transplantation are pulmonary complications (including infection, rejection, and functional limitation). More than 55 percent of the participants in the study required hospital readmission due to infection, while 24.3% experienced readmission due to rejection. Hence, the symptom of coughing is the predominant sign of presentation. These findings point to the need for health education about how to cough effectively. In the psychological domain of QOL, LTx patients have a second chance at life but are still at particular risk and face highly complex challenges, including strict medication regimens, physical rehabilitation, and the adaptation to new lifestyles and physical conditions (2, 45–47). These challenges span multiple domains and have a significant impact on both their QOL and well-being. Recipients may be surprised to find that they are not fully healthy; sometimes, they cannot even take care of themselves after surgeries. This can result in a serious deviation from preoperative expectations (48), thus expressing more concern about future health.

Our finding revealed that LTx patients with hospital readmission due to post-transplant infection were more likely to belong to the “low QOL” profile, which is consistent with previous research (2). Lung infections are strongly associated with the high risk of death (49), which may introduce an element of unpredictability and uncertainty regarding the course and outcome of lung transplantation, and thus impact their QOL (50). This finding suggested that more attention should be given to LTx patients with hospital readmission due to infection. For instance, some interventions involve closely monitoring their condition (51), implementing effective infection control measures and follow-up strategies (52), are instrumental in reducing fear of disease progression and improving QOL. Moreover, health education is important in understanding lung infection’s signs and symptoms and evolution which enables patients to seek early treatment. Meantime, taking proper precautions, such as practicing basic hand hygiene, ensuring medication compliance, and receiving vaccinations when appropriate (47), can effectively mitigate the risk of infection recurrence and enhance QOL.

In our study, we found that patients receiving bilateral lung transplantation (DLT) were more likely to be assigned to the high QOL membership compared to patients receiving single lung transplantation (SLT). This is consistent with the results of research conducted by Schaffer et al. (53). Chronic rejection, known as bronchiolitis obliterans syndrome (BOS), is a common and significant complication that affects QOL after LTx. Notably, SLT has been identified as a risk factor for the development of BOS and is associated with a lower chance of survival than BLT (54). On the other hand, Patients receiving SLT still have a native lung, the native lung exhibits compromised respiratory function because of the underlying disease process. Such a decline may potentially give rise to additional complications for SLT recipients, compounding the challenges faced by lung transplant recipients (55). On the basis of our findings, it is recommended that a dedicated rehabilitation program be tailored specifically before transplantation (56), which could contribute to a higher QOL among SLT patients. However, to date, scholars have primarily relied on individual institutional case series experiences or retrospective reviews of large LTx registries to assess the outcomes and QOL associated with SLT versus BLT. These studies have failed to identify clear criteria to determine whether SLT or BLT results in a better QOL (57).

Regarding the study’s most notable findings, it was observed that LTx patients exhibiting elevated levels of optimism, mindfulness, and a lower level of negative emotions were more likely to belong to the high QOL category than the low QOL category. These findings are supported by those of previous studies (18–20, 58). According to the transactional stress theory (59), personality characteristics, such as optimism, can impact how individuals assess situations, subsequently influencing the way they moderate stressor-induced stress responses. As such, with regard to post-transplant challenges, LTx recipients who were optimistic showed a less severe sense of threat, greater tolerance, and a higher inclination to address them than pessimists. These attitudes may help them overcome their difficulties. Besides, an optimistic disposition is believed to enhance psychological resilience, positive coping abilities, and self-efficacy, all of which play pivotal roles in enhancing LTx recipients’ ability to adapt to post-transplant challenges and contribute to their well-being. Meanwhile, optimism may also influence immune responses and neuroendocrine system modulation (60, 61), which are especially important in LTx patients with poor immunity. Thus, caregivers for “low QOL” profile patients could use encouraging words as much as possible to maintain patients’ optimistic attitude during rehabilitation sessions, which might help them cope better with the illness and more quickly improve their QOL (19). The Three Good Things (3GT) intervention is one of the most popular and effective ways to promote optimism, proven among students, healthcare workers and community-dwelling individuals (62–64). This intervention involves a simple and effective technique reflecting on and identifying three positive experiences or things that happened during the day. On one hand, it helps patients shift their attention away from negatives and toward positive experiences (65). By actively recognizing and appreciating positive events, patients can reframe their mindset and cultivate optimistic attitude. On the other hand, engaging in positive self-reflection reinforces the belief that positive outcomes could possibly occur even when facing challenges. This process enhances mental resilience and ultimately improves optimism (66). While existing literature suggests that 3GT is feasible and well-accepted, further research is needed to confirm its potential benefits in LTx patients.

In accordance with the Mindfulness-to-Meaning Theory (67), the practice of mindfulness encourages a shift from distress to a broader focus and metacognitive awareness. This shift enhances emotional regulation, facilitates disengagement from automatic thought patterns, and cultivates positive emotions (68, 69), and eventually, contributes to overall well-being, the discovery of meaning in life, and improved mental health (68). Additionally, studies on the neurophysiological mechanisms have shown that mindfulness can influence default mode (DMN), frontoparietal (FPN), and salience (SN) networks, explaining its favorable effects (70). Collectively, these results underscore the importance of establishing and nurturing positive psychological resources to sustain an improved long-term QOL among LTx recipients. Fortunately, the level of mindfulness can be improved through education (71), and studies have reported the effectiveness of mindfulness interventions in patients with lung diseases. One randomized controlled trial involving a mindfulness-based intervention group of 93 adults with asthma found post-intervention improvements in asthma-related QOL (72). In a more recent study, individuals with chronic obstructive pulmonary disease who participated in cognitive behavioral therapy support reported significant improvements in their QOL (73). Hence, future research could integrate mindfulness skills into the rehabilitation strategies of LTx recipients, particularly for the “low QOL” profile patients, and explore the optimal timing, duration, and methods to maximize its effectiveness. Furthermore, it is essential to evaluate the effectiveness of mindfulness interventions among LTx patients, including the effects of mindfulness on various outcomes, such as physical functioning, psychological well-being, and overall quality of life. By conducting comprehensive analysis of the effects of mindfulness interventions, healthcare professionals can obtain valuable guidance to develop targeted mindfulness programs and enhance the quality of life for LTx recipients.

It is worth noting that negative emotions in our study were associated with profile membership, whereas positive emotions did not display such influence. He et al. (74) have corroborated this observation, highlighting the negative emotions, rather than positive emotions, exhibits a statistically significant correlation with an individual’s QOL. In addition, a study (75) conducted in Belgium revealed that patients exhibited heightened neural activation in somatosensory regions during the observation of negative stimuli as opposed to neutral and positive stimuli, as a result of the activation of somatosensory and nociceptive brain patterns. This observation provides a more direct explanation for the pronounced adverse effects of the negative emotions on patients. Consequently, it is suggested to dynamically assess negative emotions during group interventions and regularly organize scientifically oriented activities that facilitate emotional venting in individuals experiencing negative emotions. Except for facilitating the release of emotions, it is important to approach negative emotions with the right mindset. According to the ABC theory of emotion proposed by Ellis (76), not the event itself, but the beliefs or cognitions about the event trigger emotional responses. Behavioral cognitive therapy (CBT) has demonstrated its efficacy in reducing negative emotions among heart transplant recipients (77). By decreasing negative emotion and recruiting many of the neuropsychological subcomponents that support reappraisal, patients can develop healthier strengthened emotion-regulation skills and adopt more positive responses (78). Thus, Healthcare staffs can customize CBT strategies to address the unique needs of two distinct latent profiles of QOL among lung transplant patients. Furthermore, we recommend further studies with a larger sample size to elucidate the relationship between emotional predisposition and QOL profiles and to explore the reasons for the relatively weak association observed between positive emotions and QOL category.

## Strengths and limitations

This study included using the LPA to identify QOL profiles (“low QOL” and “high QOL”), and investigated their roles of psychosocial indictors (e.g., optimism, mindfulness, and negative emotions) in QOL profile memberships among LTx patients. This approach provides valuable insights into the associations between psychosocial variables and QOL profiles, and supports the need to develop tailored and individual post-transplant interventions. However, this study had some limitations. First, this study adopted a cross-sectional design, thereby precluding the establishment of causal relationships between the study variables. Longitudinal studies are needed to explore the classes of QOL trajectories among LTx by latent growth mixture model analysis. Second, we enrolled few LTx patients within 3 months, who may not have reached a stable baseline in terms of their QOL, potentially introducing bias. Third, besides optimism, mindfulness, and negative emotions, there are other factors influencing patients’ QOL. Hence, more variables (e.g., functional status, number of hospitalizations, home versus hospital status, post-transplant co-morbidities, resilience, social support) should be considered in subsequent studies.

## Conclusion

This study examined two distinct QOL profiles in LTx patients: “low QOL” and “high QOL.” Within Chinese lung transplant recipients, we have identified that optimism, mindfulness, negative emotions, and type of operation were associated with QOL profiles. Our research findings could help develop targeted and feasible improvement programs for post-lung transplant patients to enhance their long-term QOL.

## Data availability statement

The original contributions presented in the study are included in the article/Supplementary material, further inquiries can be directed to the corresponding authors.

## Ethics statement

The studies involving humans were approved by the Ethics Committee of Guangzhou Medical University (L202210008). The studies were conducted in accordance with the local legislation and institutional requirements. The participants provided their written informed consent to participate in this study.

## Author contributions

LS: Conceptualization, Data curation, Investigation, Writing – original draft, Writing – review & editing, Validation. QL: Data curation, Methodology, Writing – original draft. CL: Conceptualization, Methodology, Writing – review & editing. YZ: Data curation, Funding acquisition, Project administration, Resources, Supervision, Validation, Writing – review & editing. DH: Data curation, Project administration, Resources, Supervision, Writing – review & editing. CJ: Data curation, Resources, Supervision, Writing – review & editing. HC: Project administration, Supervision, Validation, Writing – review & editing. TW: Validation, Visualization, Writing – review & editing. JiaC: Validation, Visualization, Writing – review & editing. WT: Investigation, Project administration, Writing – review & editing. CM: Methodology, Writing – review & editing. YM: Project administration, Validation, Writing – review & editing. JinC: Project administration, Validation, Writing – review & editing.
