# Supplementary material for: Quality of life and its association with predictors in lung transplant recipients: a latent profile analysis
Source: Front Public Health. 2024 Apr 29;12:1355179. doi: 10.3389/fpubh.2024.1355179 (PMC11089158; doi:10.3389/fpubh.2024.1355179)
Supplement: Supplementary file 1 [file Table_1.docx]

Supplementary Materials

**Table S1 Correlation analyses between the QOL subscales**

| **Variables** | **1** | **2** | **3** | **4** | **5** | **6** | **7** | **8** | **9** | **10** | **11** |
| --- | --- | --- | --- | --- | --- | --- | --- | --- | --- | --- | --- |
| 1-lung symptom | 1 |  |  |  |  |  |  |  |  |  |  |
| 2-cough | .36^**^ | 1 |  |  |  |  |  |  |  |  |  |
| 3-swallow | .45^**^ | .37^**^ | 1 |  |  |  |  |  |  |  |  |
| 4-appetite | .36^**^ | .28^**^ | .40^**^ | 1 |  |  |  |  |  |  |  |
| 5-gastric | .34^**^ | .32^**^ | .38^**^ | .35^**^ | 1 |  |  |  |  |  |  |
| 6-diarrhea | .30^**^ | .28^**^ | .31^**^ | .23^**^ | .39^**^ | 1 |  |  |  |  |  |
| 7-worry about future health | .44^**^ | .33^**^ | .26^**^ | .33^**^ | .29^**^ | .24^**^ | 1 |  |  |  |  |
| 8-anxiety | .50^**^ | .38^**^ | .40^**^ | .46^**^ | .44^**^ | .43^**^ | .56^**^ | 1 |  |  |  |
| 9-general QOL | .41^**^ | .32^**^ | .26^**^ | .44^**^ | .21^**^ | .27^**^ | .53^**^ | .54^**^ | 1 |  |  |
| 10-sexual problems | .35^**^ | .26^**^ | .22^**^ | .15 | .31^**^ | .33^**^ | .43^**^ | .49^**^ | .36^**^ | 1 |  |
| 11-cognitive limitations | .42^**^ | .33^**^ | .26^**^ | .28^**^ | .28^**^ | .31^**^ | .35^**^ | .50^**^ | .52^**^ | .23^**^ | 1 |

*Note：*^*^*P*＜0.05，^**^*P*＜0.001

**Table S2** Profiles of QOL in demographic and clinical characteristics among LTx recipients（n=173）

|  | “Low QOL” profile | “High QOL” profile | *P* |
| --- | --- | --- | --- |
| Age(years) | 56.74±13.25 | 56.27±13.07 | 0.83 |
| Gender |  |  | 0.56 |
| Male | 44 | 9 |  |
| Female | 95 | 25 |  |
| Education level |  |  | 0.85 |
| Primary school or below | 8 | 14 |  |
| Junior | 12 | 35 |  |
| High school | 12 | 23 |  |
| College or above | 21 | 48 |  |
| Marital status |  |  | 0.49 |
| Married | 51 | 117 |  |
| Unmarried | 4 | 1 |  |
| Monthly income (Chinese Yuan) |  |  | 0.15 |
| ＜3000 | 12 | 12 |  |
| 3000-5999 | 18 | 38 |  |
| 6000-8999 | 11 | 26 |  |
| 9000-11999 | 5 | 15 |  |
| ≥12000 | 7 | 29 |  |
| Diagnostic indication for transplant |  |  | 0.74 |
| Pulmonary fibrosis | 25 | 49 |  |
| Obstructive lung disease | 14 | 35 |  |
| Others | 14 | 36 |  |
| Time since lung transplant |  |  | 0.24 |
| ＜3months | 4 | 11 |  |
| 3-6months | 8 | 9 |  |
| 6months-1year | 6 | 10 |  |
| 1-3years | 23 | 51 |  |
| ＞3years | 12 | 39 |  |
| **Type of operation** |  |  | **0.02** |
| Single-lung transplantation | 28 | 26 |  |
| Bilateral lung transplantation | 40 | 80 |  |
| **BMI (kg/m^2^)** | **20.26±3.29** | **21.90±3.64** | **0.01** |
| Use of ECMO |  |  | 0.25 |
| Yes | 23 | 30 |  |
| No | 41 | 79 |  |
| Unplanned readmissions within 30 days |  |  | 0.50 |
| No | 40 | 96 |  |
| Yes | 13 | 24 |  |
| **Infection** |  |  | **＜0.001** |
| No | 11 | 61 |  |
| Yes | 42 | 59 |  |
| Rejection |  |  | 0.23 |
| No | 37 | 94 |  |
| Yes | 16 | 26 |  |
| Preoperative hypertension |  |  | 0.53 |
| No | 44 | 104 |  |
| Yes | 9 | 16 |  |
| Preoperative diabetes |  |  | 0.12 |
| No | 42 | 106 |  |
| Yes | 11 | 14 |  |
| length of post-transplant hospital stays(days) | 35.23±18.78 | 29.67±13.07 | 0.11 |
| The duration of ICU stays (days) | 18.96±24.78 | 15.06±13.59 | 0.18 |
| **Mindfulness** | **49.94±7.52** | **59.69±8.67** | **＜0.001** |
| **Optimism** | **15.79±3.89** | **19.73±3.21** | **＜0.001** |
| **Positive emotion** | **28.74±5.45** | **34.32±6.73** | **＜0.001** |
| **Negative emotion** | **22.26±6.50** | **16.97±5.26** | **＜0.001** |

Note. Boldface indicates the significantly variables.
